# Supplementary material for: Combining Mass Cytometry Data by CyTOFmerge Reveals Additional Cell Phenotypes in the Heterogeneous Ovarian Cancer Tumor Microenvironment: A Pilot Study
Source: Cancers (Basel). 2023 Oct 23;15(20):5106. doi: 10.3390/cancers15205106 (PMC10605295; doi:10.3390/cancers15205106)
Supplement: Supplementary file 1 [file cancers-15-05106-s001.zip › cancers-2628458-supplementary.pdf]

Supplementary Material

Combining Mass Cytometry Data by CyTOFmerge Reveals Additional Cell Phenotypes in the Heterogeneous Ovarian Cancer Tumor Microenvironment: A Pilot Study

Liv Cecilie Vestrheim Thomsen, Katrin Kleinmanns, Shamundeeswari Anandan, Stein-Erik Gullaksen, Tamim Abdelaal, Grete Alrek Iversen, Lars Andreas Akslen, Emmet McCormack and Line Bjørge

Supplementary tables

Table S1. Tumor dissociation methods

Patient	Tumor dissociation method
P1	Coll
P2	Mil 2 h
P3	Coll+Dis+Try
P4	Coll+Try
P5	Mil 2 h
P6	Mil 2 h
P7	Mil 1 h
P8	Coll
P9	Coll
P10	Mil 2 h

Coll: collagenase; Mil: Miltenyi; Dis: Dispase; Try: TrypLE

Described in detail in: Anandan S, Thomsen LCV, Gullaksen SE, Abdelaal T, Kleinmanns K, Skavland J, Bredholt G, Gjertsen BT, McCormack E, Bjørge L. *Phenotypic Characterization by Mass Cytometry of the Microenvironment in Ovarian Cancer and Impact of Tumor Dissociation Methods.* Cancers (Basel). 2021 Feb 11;13(4):755.
doi: 10.3390/cancers13040755. PMID: 33670410; PMCID: PMC7918057.

Table S2. The pan-tumor and pan-immune CyTOF panels

	Pan-tumor panel			Pan-immune panel		
**Tag**	**Human mAb**	**Clone**	**Dilution**	**Human mAb**	**Clone**	**Dilution**
89Y	CD45	HI30	1:1600	CD45	Hi30	1:400
141Pr	EpCAM/CD326	9C4	1:6400	EpCAM/CD326	9C4	1:3200
142Nd	CD133*	5-E3 (5E3)	1:200	CD303*	201A	1:50
143Nd	CD117	104-D2	1:50	CD117	104-D2	1:900
144 Nd				CD33*	WM53	1:100
145Nd	CD4	RPAT4	1:100	CD4	RPAT4	1:100
146Nd	CD8a	RPAT8	1:400	CD8a	RPAT8	1:200
147Sm	CD20	2H7	1:50	CD20	2H7	1:100
148Nd	CD34	581	1:3200	CD16	3G8	1:100
149Sm	TAG72*	0.N.561	1:50	CD25	2A3	1:100
150Nd	LAG-3/CD223	11C3C65	1:50			
151Eu	CD103	BER-ACT8	1:200	CD123	6H6	1:200
152Sm	CD44*	BJ18	1:400	CD95/Fas	DX2	1:200
153Eu	CD47*	CC2C6	1:12800	CD7	EH12-2H7	1:400
154Sm	TIM-3	F38-2E2	1:50	CD163	GHI/61	1:100
155Gd	PD1/CD279	EH12. 2H7	1:100	PD-1/CD279	2H7	1:200
156Gd	PDGFRB/CD140b	18A2	1:50	CD86	IT2.2	1:100
158Gd	FOLR1*	548908	1:6400	CD335*	BAB281	1:200
159Tb	PD-L1/CD274	29E.2A3	1:200	CCR7	Go43H7	1:100
160Gd	CD14	M5E2	1:200	CD14	M5E2	1:200
161Dy	CTLA-4/CD152	14D3	1:200	CTLA-4	14D3	1:200
162Dy	Foxp3	259D/C7	1:800	CD11c	Bu15	1:600
163Dy	CD56	NCAM16.2	1:12800	CD56	NCAM16.2	1:400
164Dy	CD45RO	UCHL1	1:200	CD45RO	UCHL1	1:200
165Ho	OX40/CD134*	Ber-ACT35 (ACT35)	1:100	CD127	A019D5	1:100
166Er	CD24	ML5	1:200	CD34	581	1:800
167Er	CD25*	2A3	1:50	CD27	O323	1:100
168Er	IFNg	B27	1:50			
169Tm	CD19	HIB19	1:200	CD19	HIB19	1:100
170Er	CD3	UCHT1	1:6400	CD3	UCHT1	1:1600
171Yb	AXL*	MM0098-2N33	1:200	CD62L*	DREG-56	1:100
172Yb	CD73*	AD2	1:1600	CD73*	AD2	1:1600
173 Yb				CD141	1A4	1:400
174Yb	HLA-DR	L243	1:6400	HLA-DR	L243	1:800
175Lu	aSMA*	1A4	1:6400			
176Yb	FAPalpha*	F11-24	1:800	CD1c*	L161	1:800
209Bi	CD11b	ICRF44	1:400	CD47	CC2C6	1:400

*in-house self-conjugated

Out of the 18 overlapping markers, 15 with the same metal tags in both panels are highlighted in grey. The remaining three—CD25, CD34 and CD47—are conjugated to different metal tags in the two panels.

mAb: monoclonal antibody

Table S3. Cell cluster labelling strategy. The strategy is implemented in the Astrolabe Cytometry Platform.

Compartment	Cell subset	Description
**T-cell**	NKT cell	$CD3^+$ $CD19^-$ $CD33^-$ $CD45^+$ $CD56^+$
**T-cell**	$CD8^+$ T-cell (naive)	$CD3^+$ $CD19^-$ $CD33^-$ $CD45^+$ $CD56^-$, $CD4^-$ $CD8a^+$, $CD45RO^-$, $CCR7^+$
**T-cell**	$CD8^+$ T-cell (EMRA)	$CD3^+$ $CD19^-$ $CD33^-$ $CD45^+$ $CD56^-$, $CD4^-$ $CD8a^+$, $CD45RO^-$, $CCR7^-$
**T-cell**	$CD8^+$ T-cell (effector memory)	$CD3^+$ $CD19^-$ $CD33^-$ $CD45^+$ $CD56^-$, $CD4^-$ $CD8a^+$, $CD45RO^+$, $CCR7^-$
**T-cell**	$CD8^+$ T-cell (central memory)	$CD3^+$ $CD19^-$ $CD33^-$ $CD45^+$ $CD56^-$, $CD4^-$ $CD8a^+$, $CD45RO^+$, $CCR7^+$
**T-cell**	$CD4^+$ T-cell (T-reg)	$CD3^+$ $CD19^-$ $CD33^-$ $CD45^+$ $CD56^-$, $CD4^+$ $CD8a^-$, $CD25^+$ $CD127^-$
**T-cell**	$CD4^+$ T-cell (naive)	$CD3^+$ $CD19^-$ $CD33^-$ $CD45^+$ $CD56^-$, $CD4^+$ $CD8a^-$, $CD45RO^-$, $CCR7^+$
**T-cell**	$CD4^+$ T-cell (EMRA)	$CD3^+$ $CD19^-$ $CD33^-$ $CD45^+$ $CD56^-$, $CD4^+$ $CD8a^-$, $CD45RO^-$, $CCR7^-$
**T-cell**	$CD4^+$ T-cell (effector memory)	$CD3^+$ $CD19^-$ $CD33^-$ $CD45^+$ $CD56^-$, $CD4^+$ $CD8a^-$, $CD45RO^+$, $CCR7^-$
**T-cell**	$CD4^+$ T-cell (central memory)	$CD3^+$ $CD19^-$ $CD33^-$ $CD45^+$ $CD56^-$, $CD4^+$ $CD8a^-$, $CD45RO^+$, $CCR7^+$
**T-cell**	$CD4^+$ $CD8^+$ T-cell	$CD3^+$ $CD19^-$ $CD33^-$ $CD45^+$ $CD56^-$, $CD4^+$ $CD8a^+$
**T-cell**	$CD4^-$ $CD8^-$ T-cell	$CD3^+$ $CD19^-$ $CD33^-$ $CD45^+$ $CD56^-$, $CD4^-$ $CD8a^-$
**Mast cell**	Mast Cell	$CD3^-$ $CD19^-$ $CD33^+$ $CD45^+$, $CD14^-$, $CD1c^-$ $CD16^-$ $CD117^+$ $CD141^-$
**Fibroblast**	Fibroblast	$CD45^-$, $CD56^+$
**Other immune cell**	CM^- $HLA\text{-}DR^+$	$CD3^-$ $CD19^-$ $CD33^-$ $CD45^+$ $CD56^-$, $CD123^-$ $HLA\text{-}DR^+$
**Tumor cell**	$CD45^-$ $CD56^-$ ($EpCAM^+$ $FOLR1^+$ $CD24^+$)	$CD45^-$, $CD56^-$, $CD24^+$ $EpCAM^+$ $FOLR1^+$
**Tumor cell**	$CD45^-$ $CD56^-$ ($EpCAM^+$ $FOLR1^+$ $CD24^-$)	$CD45^-$, $CD56^-$, $CD24^-$ $EpCAM^+$ $FOLR1^+$
**Tumor cell**	$CD45^-$ $CD56^-$ ($EpCAM^+$ $FOLR1^-$ $CD24^+$)	$CD45^-$, $CD56^-$, $CD24^+$ $EpCAM^+$ $FOLR1^-$
**Tumor cell**	$CD45^-$ $CD56^-$ ($EpCAM^+$ $FOLR1^-$ $CD24^-$)	$CD45^-$, $CD56^-$, $CD24^-$ $EpCAM^+$ $FOLR1^-$
**Tumor cell**	$CD45^-$ $CD56^-$ ($EpCAM^-$ $FOLR1^+$ $CD24^+$)	$CD45^-$, $CD56^-$, $CD24^+$ $EpCAM^-$ $FOLR1^+$

**Tumor cell**	CD45- CD56- (EpCAM- FOLR1+ CD24-)	$CD45^-$, $CD56^-$, $CD24^-$ $EpCAM^-$ $FOLR1^+$
**Tumor cell**	$CD45^-$ $CD56^-$ ($EpCAM^-$ $FOLR1^-$ $CD24^+$)	$CD45^-$, $CD56^-$, $CD24^+$ $EpCAM^-$ $FOLR1^-$
**Tumor cell**	$CD45^-$ $CD56^-$ ($EpCAM^-$ $FOLR1^-$ $CD24^-$)	$CD45^-$, $CD56^-$, $CD24^-$ $EpCAM^-$ $FOLR1^-$
**DC**	$CD1c^-$ $CD141^-$ DC (conventional)	$CD3^-$ $CD19^-$ $CD33^+$ $CD45^+$, $CD14^-$, $CD1c^-$ $CD16^-$ $CD117^-$ $CD141^-$
**NK cell**	NK cell ($CD56^+$ $CD16^+$)	$CD3^-$ $CD19^-$ $CD33^-$ $CD45^+$ $CD56^+$, $CD16^+$
**NK cell**	NK cell ($CD56^+$ $CD16^-$)	$CD3^-$ $CD19^-$ $CD33^-$ $CD45^+$ $CD56^+$, $CD16^-$

NK: natural killer cells; EMRA: effector memory cells re-expressing CD45RA; T-reg: regulatory T cells; DC: dendritic cells

Table S4. Distribution of the main cell populations across samples (the pan-tumor panel)

Sample name	Total cell number*	Immune cells**		Tumor cells		Fibroblasts		Linage-negative cells	
		No. of cells	Frequency (%)	No. of cells	Frequency (%)	No. of cells	Frequency (%)	No. of cells (n)	Frequency (%)
P1	48823	2163	4.43	41273	84.54	5387	11.03	15	0.03
P2	2097	1254	59.80	683	32.57	142	6.77	18	0.86
P3	39029	6838	17.52	27742	71.08	4393	11.26	56	0.14
P4	35887	5525	15.40	21309	59.38	9021	25.14	32	0.09
P5	481930	241883	50.19	230478	47.82	9463	1.96	106	0.02
P6	230211	21159	9.19	145697	63.29	62702	27.24	653	0.28
P7	97895	9032	9.23	69704	71.20	18628	19.03	531	0.54
P8	144164	34182	23.71	103201	71.59	6604	4.58	177	0.12
P9	213673	17016	7.96	172556	80.76	23931	11.20	170	0.08
P10	546534	23058	4.22	499001	91.30	24245	4.44	230	0.04

*Excluding the debris

**Including the $CD45^{+}CD11b^{+}$immune cell populations

Table S5. Distribution of the main cell populations across samples (the pan-immune panel)

Sample name	Total cell number*	Immune cells**		Tumor cells		Fibroblasts		Linage-negative cells	
		No. of cells	Frequency (%)	No. of cells	Frequency (%)	No. of cells	Frequency (%)	No. of cells	Frequency (%)
P1	3046	1770	58.1	NA	NA	NA	NA	1276	41.9
P2	1774	514	29.0	NA	NA	NA	NA	1260	71.0
P3	7053	4014	56.9	NA	NA	NA	NA	3039	43.1
P4	6282	5484	87.3	NA	NA	NA	NA	798	12.7
P5	383383	182798	47.7	NA	NA	NA	NA	200585	52.3
P6	38859	18304	47.1	NA	NA	NA	NA	20555	52.9
P7	32445	20639	63.6	NA	NA	NA	NA	11806	36.4
P8	59610	18375	30.8	NA	NA	NA	NA	41235	69.2
P9	23935	12582	52.6	NA	NA	NA	NA	11353	47.4
P10	57326	26432	46.1	NA	NA	NA	NA	30894	53.9

*Excluding the debris

**Including the $CD45^{+}CD11b^{+}$immune cell populations

Table S6. Distribution of the main cell populations across samples (the merged dataset)

Sample name	Total cell number*	Immune cells**		Tumor cells		Fibroblasts		Linage-negative cells	
		No. of cells	Frequency (%)	No. of cells	Frequency (%)	No. of cells	Frequency (%)	No. of cells	Frequency (%)
P1	103570	2695	2.6	86097	83.1	13094	12.6	1684	1.6
P2	4381	814	18.6	1286	29.4	291	6.6	1990	45.4
P3	75054	10244	13.6	52670	70.2	9525	12.7	2615	3.5
P4	80635	7727	9.6	48288	59.9	20012	24.8	4608	5.7
P5	927000	93816	10.1	417434	45.0	18127	2.0	397623	42.9
P6	543214	41895	7.7	343957	63.3	145372	26.8	11990	2.2
P7	181726	1515	0.8	128881	70.9	38308	21.1	13022	7.2
P8	281512	24550	8.7	207755	73.8	15986	5.7	33221	11.8
P9	404409	23066	5.7	328707	81.3	52636	13.0	0	0.0
P10	1073767	47619	4.4	976830	91.0	49165	4.6	153	0.0

*Excluding the debris

**Including the $CD45^+CD11b^+$immune cell populations

Table S7. Associations between phenotypes and presence of hierarchically clustered (FlowSOM) cell subsets containing ≥50 cells across patient samples (n = 141).

Dataset	Phenotype	Cell subset	logFC	P value	FDR
Pan-tumor					
	Debulking status	Fibroblasts aSMA^{lo}EpCAM^{lo}FOLR1^{hi}	-13.37	0.008	0.75
	Recurrence	EpCAM^{+}FOLR1^{-}CD24^{-}No Profile cells	-9.34	0.007	0.44
	Clinical status	CM-unassigned CD24^{hi}CD11b^{hi}cells	5.79	0.003	0.33
Pan-immune					
	Debulking status	Conventional CD1c^{-}CD141^{-}dendritic cells CD11c^{lo}HLA-DR^{lo}CD163^{hi}	-11.95	0.0019	0.17
		Monocytes/macrophages (CD14^{-}CD16^{+}) No Profile	4.81	0.006	0.37
	Recurrence	Conventional CD1c^{-}CD141^{-}dendritic cells CD11c^{hi}HLA-DR^{lo}	7.85	7.74E-05	0.01
		Monocytes/macrophages (CD14^{-}CD16^{+}) CD45RO^{lo}CD86^{lo}HLA-DR^{hi}	9.87	0.0008	0.04
		Type 1 CD141^{+}dendritic cells No Profile	9.83	0.0008	0.04
		Monocytes/macrophages (CD14^{-}CD16^{+}) CD45RO^{hi}CD86^{lo}HLA-DR^{hi}	9.81	0.0008	0.04
		Monocytes/macrophages (CD14^{+}CD16^{+}) CD11c^{hi}CD25^{lo}CD56^{lo}	7.35	0.005	0.15
	Clinical status	Type 1 CD141^{+}dendritic cells No Profile	11.13	3.31E-05	0.01
		Conventional CD1c^{-}CD141^{-}dendritic cells CD11c^{hi}HLA-DR^{lo}	9.32	0.0001	0.01
		Monocytes/macrophages (CD14^{-}CD16^{+}) CD45RO^{hi}CD86^{lo}HLA-DR^{hi}	10.84	0.0007	0.04
		Monocytes/macrophages (CD14^{-}CD16^{+}) CD45RO^{lo}CD86^{lo}HLA-DR^{hi}	10.63	0.0009	0.04
		CD4^{+}CD8^{+}T cells CD141^{lo}CD32CD7^{hi}CD86^{lo}	6.25	0.001	0.04
		Type 2 CD1c^{+}dendritic cells No Profile	6.77	0.002	0.06
		Naive CD8^{+}T cells HLA-DR^{lo}CD127^{lo}PD1^{lo}	6.15	0.004	0.09
		Type 2 CD1c^{+}dendritic cells HLA-DR^{hi}CCR7^{lo}CD127^{hi}	5.04	0.008	0.15
Merged					
	Debulking status	EpCAM^{+}FOLR1^{-}CD24^{+}CCR7^{hi}TAG72^{hi}cells	12.56	0.008	0.51
		EpCAM^{+}FOLR1^{-}CD24^{+}CCR7l^{o}TAG72^{lo}FOXP3^{hi}cells	12.40	0.009	0.51
	Recurrence				
		NK cells (CD56^{+}CD16^{-})CD141^{hi}CD163^{hi}CD11c^{hi}	-11.71	0.0002	0.02
		NK cells (CD56^{+}CD16^{-}) No Profile	-12.59	0.0003	0.02
		NK cells (CD56^{+}CD16^{-})CD141^{lo}CD163^{lo}CD11c^{lo}	-11.83	0.0005	0.02
		NKT cells	-10.46	0.0004	0.02
		CD4^{-}CD8^{-}T cells PD1^{hi}CD127^{hi}	-9.97	0.0007	0.03
		NK cells (CD56^{+}CD16^{-})CD141^{lo}CD163^{lo}CD11c^{hi}	-9.59	0.0009	0.03
		Monocytes/macrophages (CD14^{+}CD16^{-}) No Profile	-8.56	0.001	0.03
		NK cells (CD56^{+}CD16^{+}) No Profile	-8.44	0.001	0.03
		Plasmacytoid dendritic cells CD34^{lo}CD86^{lo}CD73^{lo}	-9.79	0.002	0.04
		Type 1 CD141^{+}dendritic cells No Profile	8.90	0.005	0.07
		EpCAM^{-}FOLR1^{+}CD24^{+}cells TAG72^{lo}CD62L^{lo}CD20^{hi}	-7.72	0.005	0.07
		Type 2 CD1c^{+}dendritic cells CD127^{hi}CD56^{hi}	7.75	0.005	0.07

	Effector memory CD4^{+}T cells CTLA4^{lo}PD1^{hi}OX40^{hi}	-6.58	0.006	0.08
	Central memory CD4^{+}T cells PD1^{lo}CD127^{hi}OX40^{hi}	-6.81	0.007	0.08
Clinical status	EpCAM^{-}FOLR1^{+}CD24^{+}cells TAG72^{lo}CD62L^{lo}CD20^{lo}	-14.29	0.0005	0.06
	CM-unassigned cells CD8^{lo}CD16^{hi}	-7.51	0.0006	0.06
	CM-unassigned cells CD8^{lo}CD16^{lo}CD32CD7^{lo}	-10.80	0.002	0.09
	EpCAM^{-}FOLR1^{+}CD24^{+}cells TAG72^{lo}CD62L^{lo}CD20^{hi}	-11.04	0.003	0.09
	EpCAM^{-}FOLR1^{+}CD24^{+}cells TAG72^{lo}CD62L^{hi}	-11.31	0.003	0.09
	NK cells (CD56^{+}CD16^{-}) CD141^{hi}CD163^{hi}CD11c^{hi}	-11.44	0.003	0.09
	NK cells (CD56^{+}CD16^{-}) No Profile	-12.32	0.004	0.09
	NK cells (CD56^{+}CD16^{-}) CD141^{lo}CD163^{lo}CD11c^{lo}	-11.57	0.004	0.09
	Plasmacytoid dendritic cells CD34^{lo}CD86^{lo}CD73^{lo}	-11.89	0.004	0.09
	NKT cells	-10.19	0.005	0.10
	EpCAM^{-}FOLR1^{+}CD24^{+}cells TAG72^{hi}	-9.98	0.006	0.11
	CD4^{-}CD8^{-}T cells PD1^{hi}CD127^{hi}	-9.66	0.009	0.14
	CM^{-}HLADR^{+}cells CD1c^{lo}CD163^{lo}CD4^{lo}	-11.30	0.010	0.14
	NK cells (CD56^{+}CD16^{-}) CD141^{lo}CD163^{lo}CD11c^{hi}	-9.32	0.010	0.14

Associations with p-values < 0.01 are shown.

logFC: logarithmically transformed fold change, FDR: false discovery rate, NK: natural killer, EpCAM: epithelial cellular adhesion molecule**,** FOLR1: folate receptor alpha, HLA-DR: major histocompatibility complex (MHC) II cell surface receptor

Supplementary figures

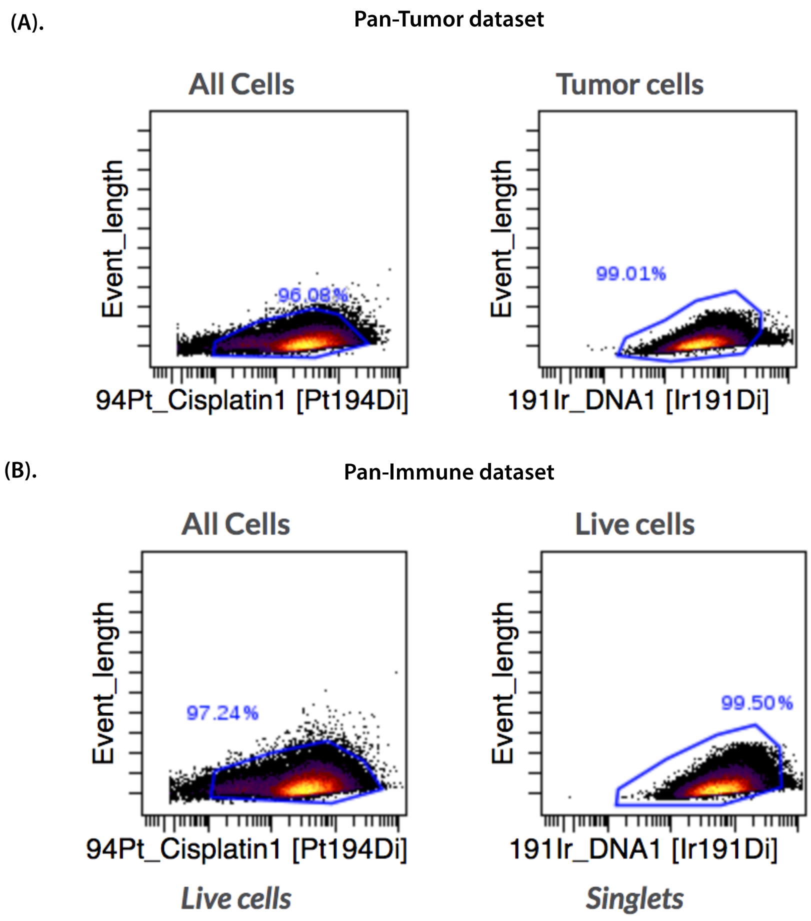

Figure S1. Data clean up: gating strategy illustration. (A) Pan-tumor dataset, (B) Pan-immune dataset

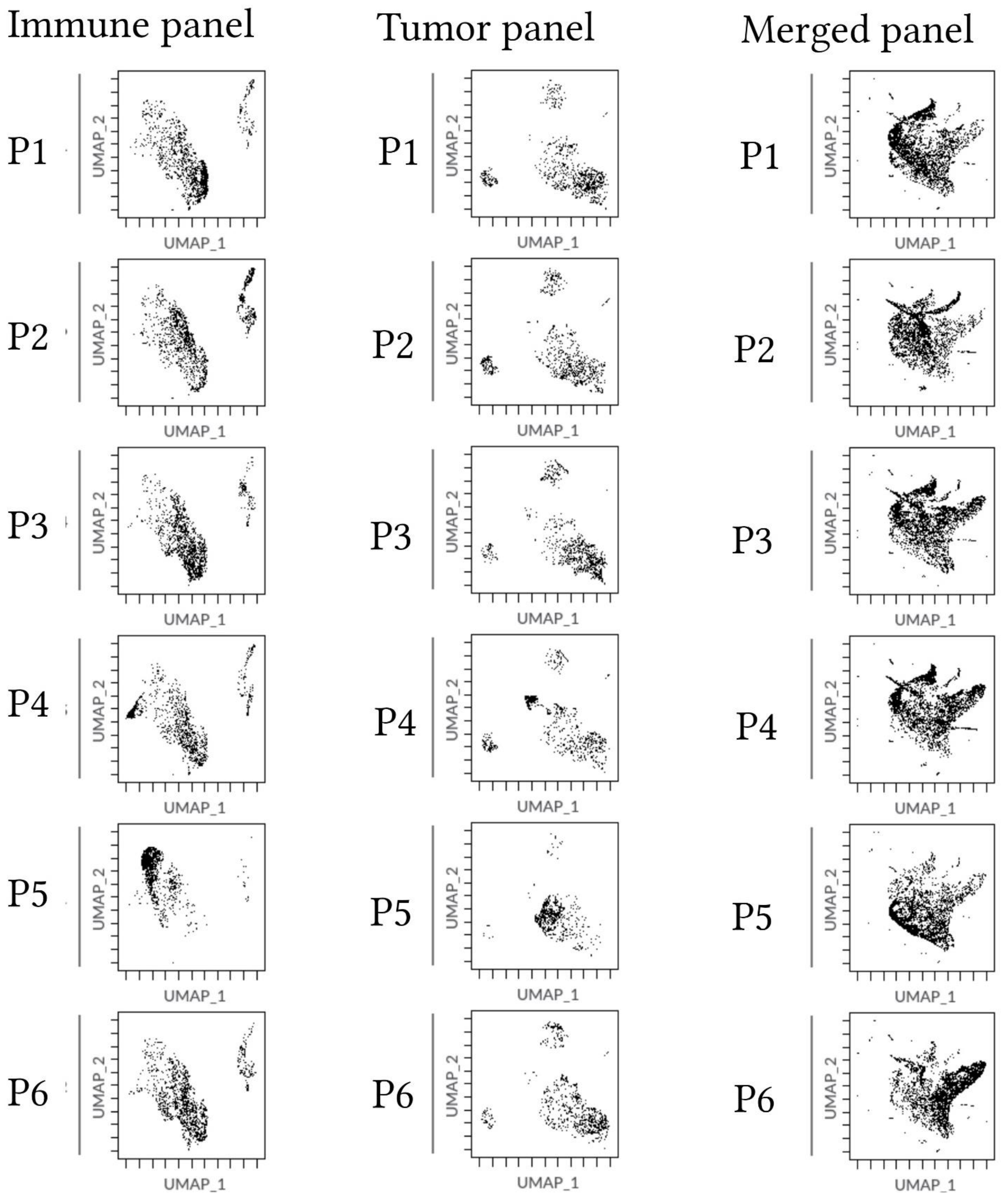

Immune panel
Tumor panel
Merged panel
P1
P2
P3
P4
P5
P6
UMAP_2
UMAP_1

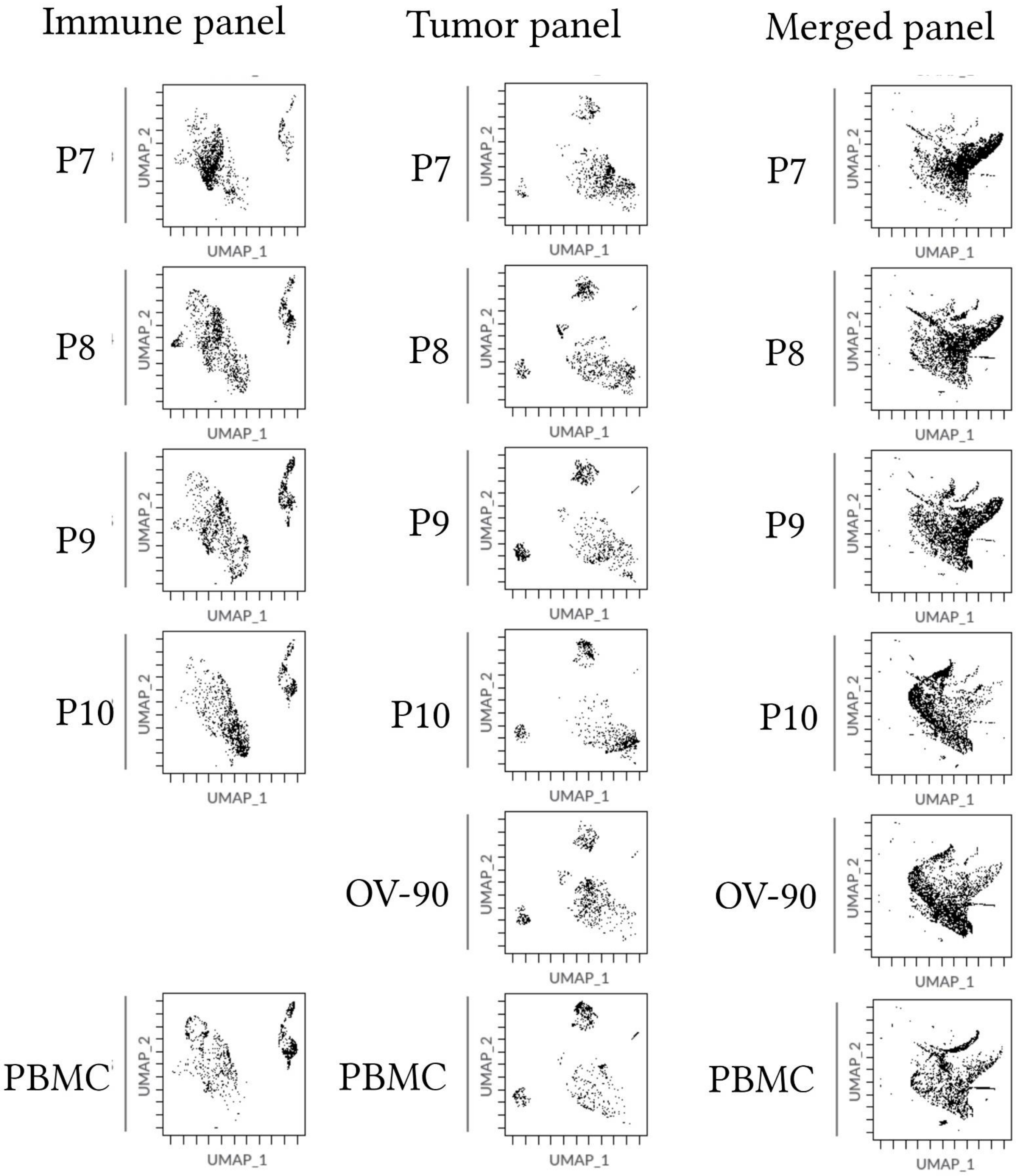

Figure S2. UMAP plots depicting the presence of and distribution of the CD45+ cell population in all patients and control samples. The three datasets were examined by the same gating strategy in Cytobank (Quality Control).

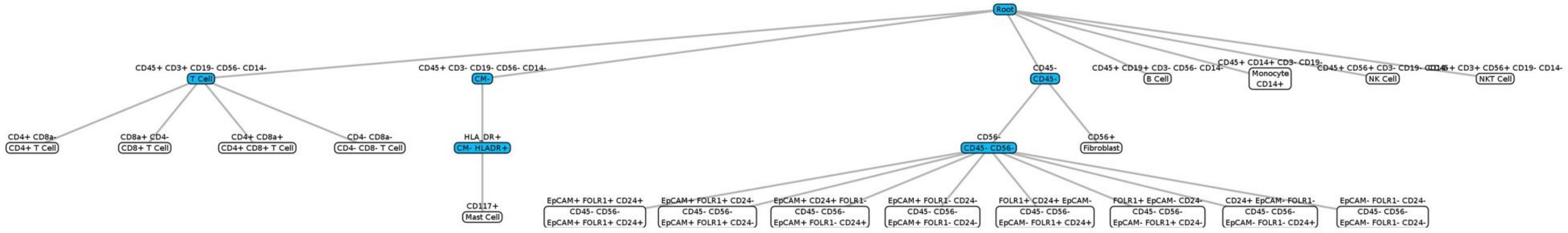

Figure S3. Astrolabe labeling hierarchy for the tumor panel (custom built)

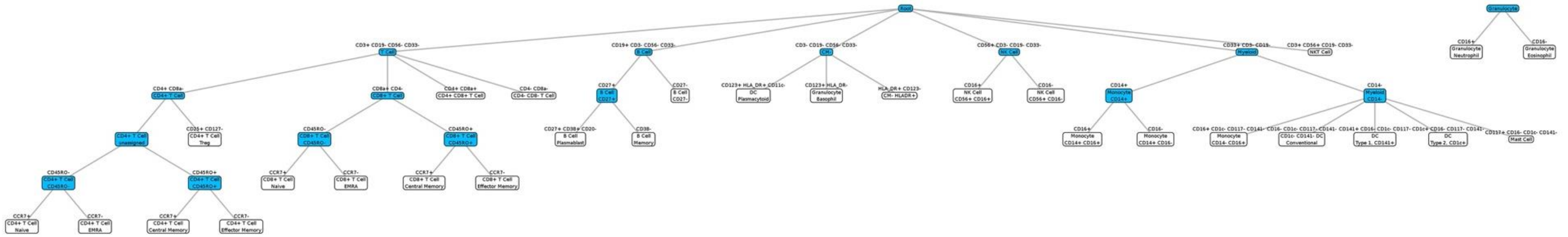

Figure S4. Astrolabe labeling hierarchy for the immune panel (build v.19.12).

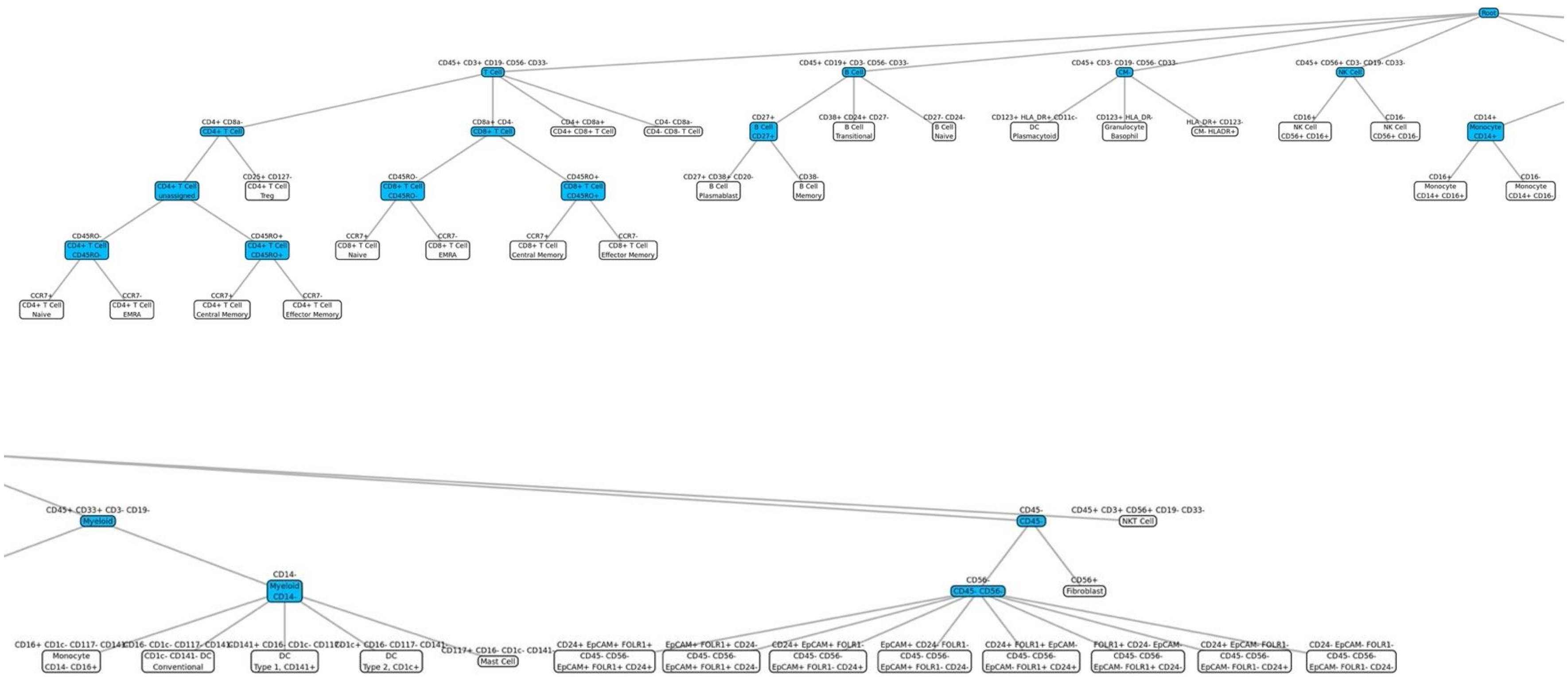

Figure S5. Astrolabe labeling hierarchy for the merged panel. Astrolabe Build v.19.12 was used for immune cells, and the hierarchy was custom built for the CD45 negative cells.

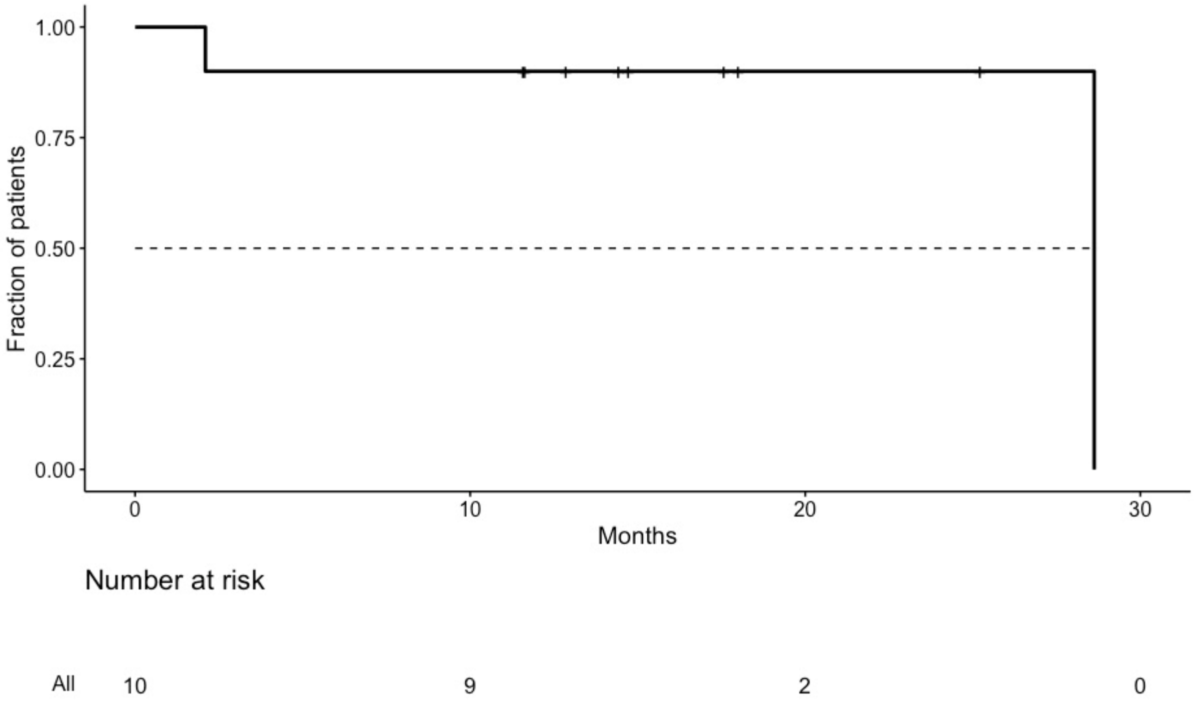

Figure S6. Overall survival. Kaplan-Meier curve illustrating the overall survival of the cohort. The number of months is represented on the X axis, and the number of patients alive is presented as a fraction of the total study cohort on the Y axis. The total number of patients corresponding to each survival period is presented in the risk table below the X axis. The tick marks demonstrate censoring at the data cut-off time point. The dotted line indicates the time point when 50% of patients are alive.

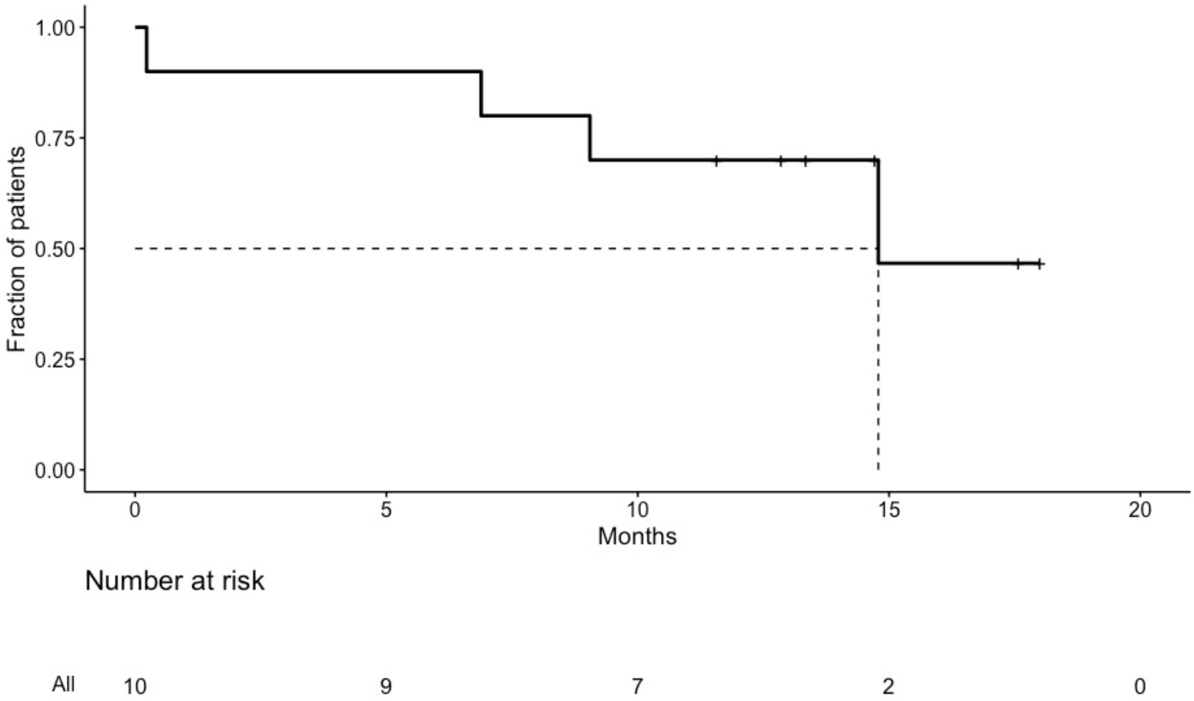

Figure S7. Progression-free survival. Kaplan-Meier curve illustrating the number of patients who developed recurrent disease. The number of months of progression-free survival is represented on the X axis, and the fraction of patients of the total cohort who did not have recurrent disease is represented on the Y axis. The total number of patients corresponding to each time point is shown in the risk table below the X axis. The tick marks demonstrate censoring at the data cut-off time point. The dotted line indicates the time point when 50% of patients are alive.

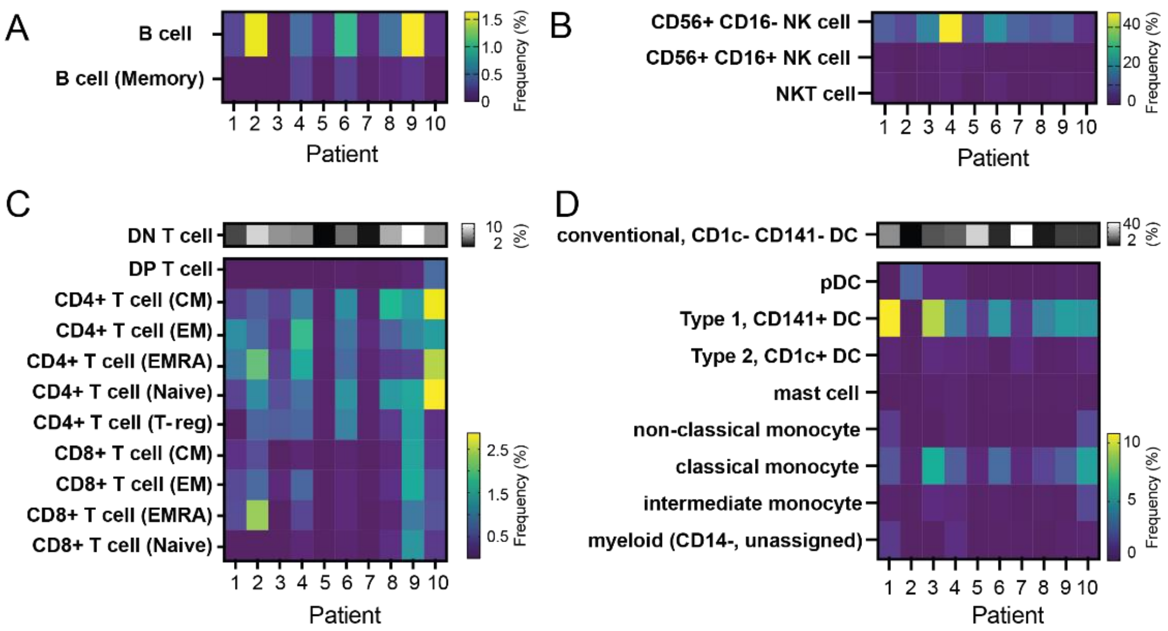

Figure S8. Heatmaps illustrating interpatient differences in the main immune cell populations of the patients' tumor immune microenvironment. The immune cell subsets of (A) B cells, (B) NK cells, (C) T cells, and (D) myeloid immune cells detected in the tumors are presented on the Y axis, and individual patients are indicated along the X axis. The double negative T cell and the conventional $CD1c^{-}CD141^{-}$DC immune cell populations have been plotted separately with an adapted heatmap.

DN: CD4/CD8 double negative; DP: CD4/CD8 double positive

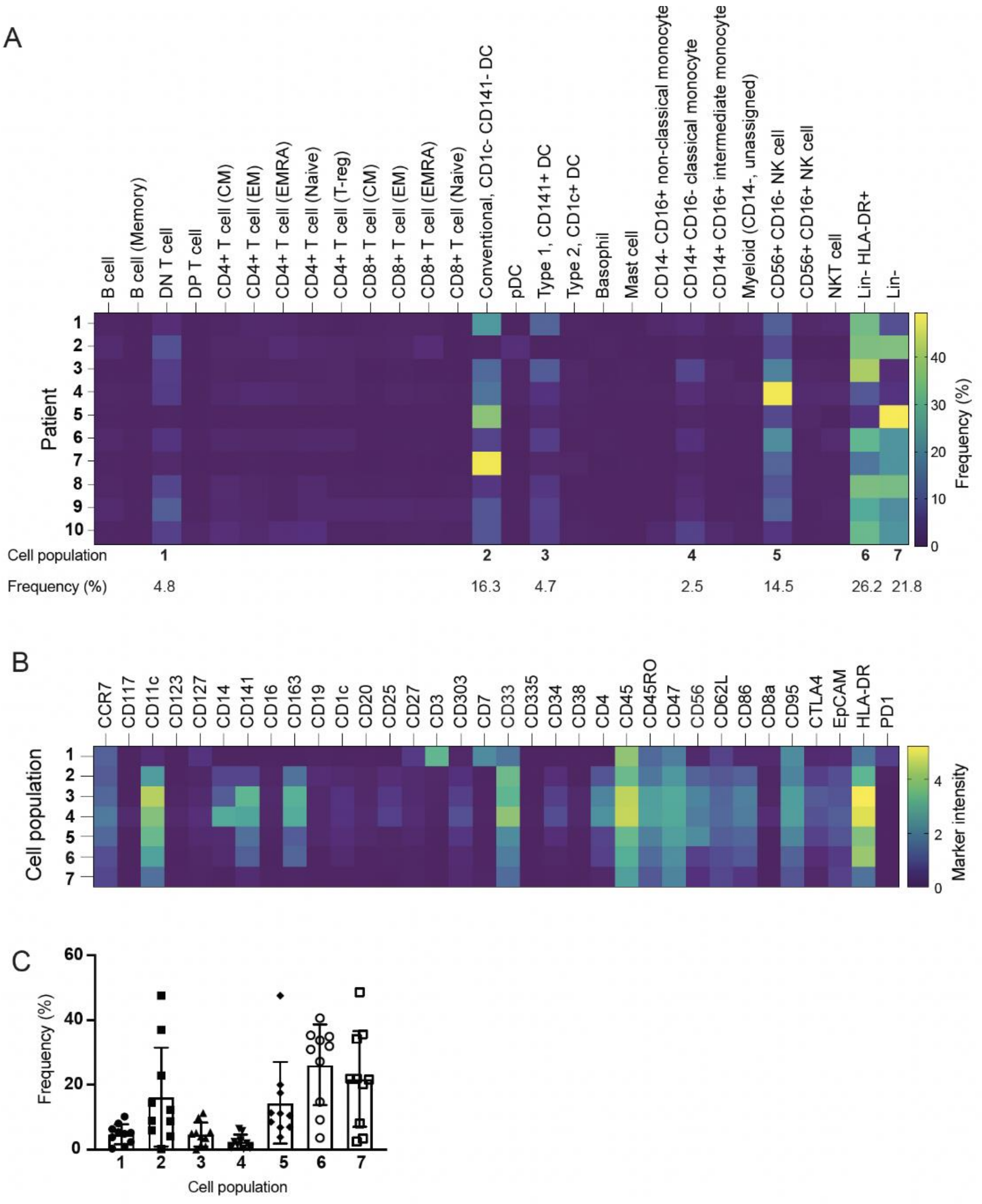

Figure S9. The seven most frequent immune cell populations in the patient samples. Frequencies are shown for the seven most common immune cell populations and expression intensity of their markers in the patient samples; 1: double-negative (DN) T cells, 2: conventional (CD1c-CD141-) dendritic cells (DC), 3: Type 1 (CD141+) DC, 4: CD14+CD16- classical monocytes/macrophages, 5: CD56+CD16- natural killer (NK) cells, 6: HLA-DR+ lineage-negative cells, 7: lineage-negative cells

(A) Heatmap depicting the frequencies of all the cell populations for each of the ten patients, with the percentages of the seven most common ones across all the samples provided separately in the row below. (B) Heatmap illustrating the marker expression intensity (columns) for each of the seven most frequent cell subsets. Marker intensity at the baseline is set to 0. (C) Boxplot showing the distribution of the frequency (Y axis) of the seven most common cell populations (1–7, X axis) for the ten patient samples.

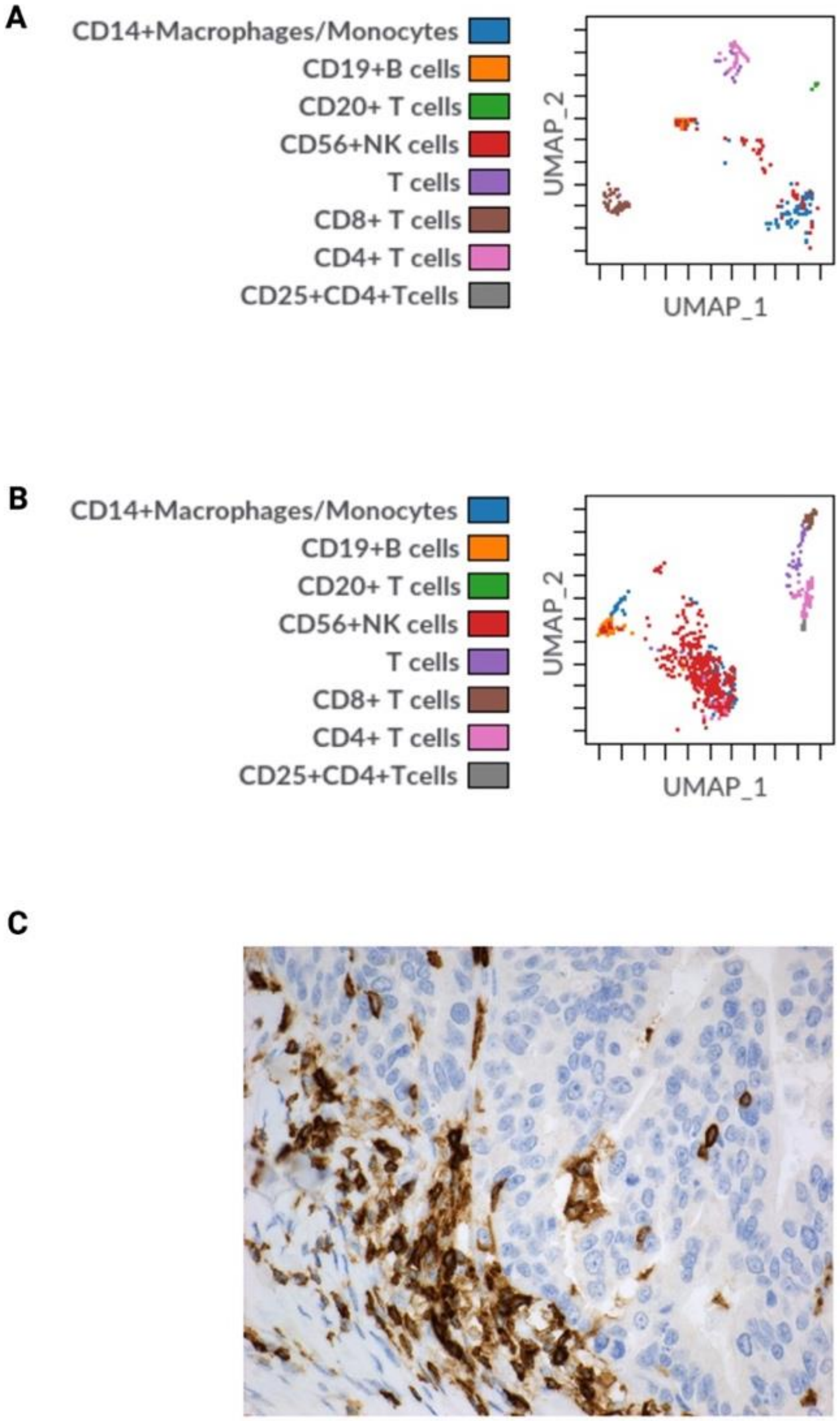

Figure S10. Distribution of immune cell populations for patient P5. Panels A and B depict the distribution of immune cell populations determined according to UMAP analysis of the pan-tumor panel (A) and the pan-immune panel (B). The same gating strategy was applied to both panels to identify different cell subsets, which are color coded. (C) When the sample was examined by immunohistochemistry with an antibody against CD45, the leukocytes stained brown, while the other cells remained unstained.
